# Supplementary material for: PoxiPred: An Artificial-Intelligence-Based Method for the Prediction of Potential Antigens and Epitopes to Accelerate Vaccine Development Efforts against Poxviruses
Source: Biology (Basel). 2024 Feb 17;13(2):125. doi: 10.3390/biology13020125 (PMC10887159; doi:10.3390/biology13020125)
Supplement: Supplementary file 1 [file biology-13-00125-s001.zip › biology-2814629-supplementary.pdf]

Supplementary Table S1- List of 25 poxviruses along with proteome IDs and number of proteins

S. No.	Name of the Virus	Proteome ID	Number of Proteins
1	Lumpy skin Disease virus	UP000315615	156
2	Myxoma virus	UP000291359	158
3	Orf virus	UP000693994	130
4	Sealpox virus	UP000202998	119
5	Pseudocowpox virus	UP000117145	125
6	Sheeppox virus	UP000318262	129
7	Ectromelia virus	UP000130118	180
8	Squirrel pox virus	UP000144311	141
9	Camelpox virus	UP000107153	261
10	Yaba monkey tumor virus	UP000008596	140
11	Tanapox virus	UP000099606	155
12	Swinepox virus	UP000000871	146
13	Turkeypox virus	UP000142477	170
14	Taterapox virus	UP000139570	220
15	Vaccinia virus	UP000000344	218
16	Variola Virus	UP000002060	198
17	Cowpox virus	UP000097203	214
18	Horsepox virus	UP000111173	228
19	Monkeypox virus	UP000516359	183
20	Molluscum contagiosum virus subtype	UP000000869	163
21	Fowlpox virus	UP000150838	232
22	Bovine papular stomatitis virus	UP000104372	130
23	Canarypox virus	UP000168164	322
24	Volepox virus	UP000203649	204

25	Goatpox virus	UP000134642	149

Supplementary Table S2 – Antigen classification performance

	Accuracy	Precision	Recall	F1 Score
Random Forest	0.51	0.56	0.40	0.47
Support Vector Machines	0.65	0.67	0.71	0.69
Logistic Regression	0.63	0.65	0.67	0.66
Gradient Boosting	0.49	0.53	0.42	0.47
Extreme Gradient Boosting	0.54	0.57	0.51	0.54
K-Nearest Neighbors	0.62	0.63	0.69	0.66

Supplementary Table S3 – T cell epitopes classification performance

	Accuracy	Precision	Recall	F1 Score
Random Forest	0.66	0.67	0.68	0.67
Support Vector Machines	0.53	0.55	0.38	0.45
Logistic Regression	0.53	0.54	0.46	0.50
Gradient Boosting	0.67	0.70	0.61	0.65
Extreme Gradient Boosting	0.68	0.69	0.67	0.68
K-Nearest Neighbors	0.57	0.65	0.32	0.43
